# Anger expression, violent behavior, and symptoms of depression among male college students in Ethiopia

**DOI:** 10.1186/1471-2458-9-13

**Published:** 2009-01-12

**Authors:** Dale J Terasaki, Bizu Gelaye, Yemane Berhane, Michelle A Williams

**Affiliations:** 1 Department of Epidemiology, Multidisciplinary International Research Training Program, University of Washington School of Public Health and Community Medicine, Seattle, Washington, USA; 2 Addis Continental Institute of Public Health, Addis Ababa, Ethiopia; 3 Department of Community Medicine, Addis Ababa University Medical School, Addis Ababa, Ethiopia

## Abstract

**Background:**

Depression is an important global public health problem. Given the scarcity of studies involving African youths, this study was conducted to evaluate the associations of anger expression and violent behavior with symptoms of depression among male college students.

**Methods:**

A self-administered questionnaire was used to collect information on socio-demographic and lifestyle characteristics and violent behavior among 1,176 college students in Awassa, Ethiopia in June, 2006. The questionnaire incorporated the Spielberger Anger-Out Expression (SAOE) scale and symptoms of depression were evaluated using the Patient Health Questionnaire (PHQ-9). Multivariable logistic regression procedures were used to calculate adjusted odds ratios (OR) and 95% confidence intervals (95%CI).

**Results:**

Symptoms of depression were evident in 23.6% of participants. Some 54.3% of students reported committing at least one act of violence in the current academic year; and 29.3% of students reported high (SAOE score ≥ 15) levels of anger-expression. In multivariate analysis, moderate (OR = 1.97; 95%CI 1.33–2.93) and high (OR = 3.23; 95%CI 2.14–4.88) outward anger were statistically significantly associated with increased risks of depressive symptoms. Violent behavior was noted to be associated with depressive symptoms (OR = 1.82; 95%CI 1.37–2.40).

**Conclusion:**

Further research should be conducted to better characterize community and individual level determinants of anger-expression, violent behavior and depression among youths.

## Background

Depression is an important global public health problem, in part, because of its high lifetime prevalence [1], and its association with poverty, malnutrition, and debilitating chronic disorders including angina, arthritis, diabetes, chronic headaches and migraines [1,2]. Several investigators have documented associations between depression and outward anger expression [3-5]. Collectively, available evidence suggest that aggressive, outward violent behavior is associated with an increased risk of symptoms of depression [6,7]. Motivated, in part, by the high prevalence of violence among youths [8] and increasing awareness of the increasing burden of anxiety and depressive disorders among youths [9], investigators have begun to evaluate associations of anger coping styles, violent behavior, and symptoms of depression among youths [10-13]. Goodwin [10], in her analysis of data from a health behavior survey of US school-aged children, noted that outward anger expression was associated with an almost 3-fold increased risk of feelings of depression among boys. Similar positive associations between anger expression with symptoms of depression have been reported by others including those who studied male Japanese students [14], as well as Turkish and American female students [13]. In Iceland, Gudlaugsdottir and colleagues [15] documented associations between violent behavior and negative life events. Additionally, Orpinas et al [16] noted that students exhibiting violent behaviors in schools (e.g., involved in physical fights, carried a weapon to school) were more likely to have symptoms of depression than students not exhibiting such behaviors. To date, few studies evaluated associations of violent behavior, anger coping styles, and symptoms of depression among African youths. Available data, although scarce, from studies conducted among Ethiopia youths [17-19] suggest high frequencies of outward anger expression among male college students [19], and high frequencies of feelings of depression among secondary school students [17,18]. It should be noted that most studies that have evaluated anger expression, violent behavior and depression among college students have primarily been focused on North American and European populations. The extent to which, if at all, inferences from prior studies may be generalizable to African college students is unknown. Notably, investigators have reported that social environments and cultural backgrounds are important determinants of how depression is experienced and expressed in psychological, emotional, or physical terms [20-23]. Given the scarcity of studies involving African youths and the need for evidence based cross-cultural understanding of expression of emotions, we used data from a previously completed survey [19] to evaluate the extent to which outward anger expression and violent behavior are associated with symptoms of depression among Ethiopian male college students.

## Methods

### Sampling Procedure and Study Population

This cross sectional study was conducted in Awassa, Ethiopia, 275 kilometers south of the country's capital, Addis Ababa, during the months of June and July, 2006. The study location was determined by the investigative team. The goal was to study a population in Ethiopia that had not been studied before. Eight private and public colleges and one university (hereinafter referred to as "colleges") were selected for participation in the survey. A total of 50 departments were identified from among the nine colleges. The departments included diverse areas of study such as: laboratory sciences, clinical nursing, pharmacy, law, accounting, secretarial science, natural science, social science, and fine arts. From these 50 departments, 17 departments were selected randomly to get a sufficient number of departments that would yield enough students to meet our sample size requirements. Only male students within the 17 departments selected were eligible for inclusion in the survey.

### Data Collection and Management

Briefly, this analysis was based on information collected from a self-administered questionnaire. Depressive symptoms were assessed using questions from the PHQ-9 Quick Depression Assessment tool [24]. The PHQ-9 consists of nine questions based on the *DSM-IV *criteria for a major depressive episode. Each of the questions asks participants to select the frequency of the depressive symptoms that they experienced during the current academic year. Scores for each of the 9 items range from 0 ('not at all') to 3 ('nearly every day'). Anger expression was assessed using the Spielberger Anger-Out Expression Scale [25]. The 8-item scale evaluates respondents' coping methods, particularly outward expressive behavior, when angry. Students were asked about the following outward behaviors: conveying anger feelings, making sarcastic remarks, slamming doors, arguing with others, striking out when infuriated, saying nasty things, losing their temper, and expressing their feelings if annoyed during the current academic year. In light of concerns about participant burden, other subscales from the Spielberger instrument were not used in this study. The Spielberger Anger-Out Expression Scale has been successfully used among college students and prior investigators reported high degrees of validity and internal consistency (alpha ranging from 0.80 to 0.95) among college students [25-27].

Violent behavior was defined as the intentional use of physical force, threatened or actual, against another person (regardless of gender), that has a high likelihood of resulting in physical or psychological harm [8]. The questionnaire contained specific, objective descriptions of eight physically violent behaviors. These questions have been used previously to assess violent behavior among young students [15]. Slapping, throwing things, pushing/shoving, kicking, dragging, strangling, choking, burning, threatening with a weapon or using a weapon (gun, knife or other object) were classified as physically violent behaviors. Negative life events were assessed by using an 18-item questionnaire. Participants were asked to indicate whether potentially stressful events, such as breaking up with a girlfriend had occurred in the current academic year. Those responding in the affirmative were asked to indicate the impact, ranging from very negative to very positive [28,29] of the event. These questions have been used previously to estimate negative life events in Ethiopia, though psychometric properties of this instrument have not yet been published [19].

Information concerning socio-demographic and lifestyle characteristics, including use of khat leaves (*Catha edulis*), a natural stimulant with amphetamine-like effects, commonly used recreationally in Ethiopia [30], was also collected. The questionnaire was first written in English and then translated to Amharic, Ethiopia's primary language. The Amharic version of the questionnaire was then pre-tested among students in a private college in Addis Ababa.

All students from selected departments were invited to a pre-selected area, typically the college's auditorium, where the overall objective of the research project was described and discussed. Students interested in participating in the study were invited to complete the study questionnaire. All students invited to participate in the survey elected to do so. All completed questionnaires were anonymous and no personal identifiers were used. Information from questionnaires were double entered into EPI INFO (Version 3.3.2), a public access software made available from the US Centers for Disease Control and Prevention. Entered data were exported to SPSS (version 13.0, SPSS Inc. Chicago, IL, USA) for statistical analysis. All study procedures were approved by the Institutional Review Boards of Addis Ababa University, Awassa Health Sciences College, and Human Subjects Division of the University of Washington.

### Variable specifications and statistical analyses

A total of 1,176 male students were included in this analysis. Students' depression status for the academic year was defined using the information that was collected from the PHQ questions. The scoring system described by Kroenke et al [31] was used to categorize students into the following groups: no depression (scores <10); depression of any severity (scores ≥ 10). An overall anger expression score was computed by summing responses for each of the eight items on the scale. Scores were categorized *a priori *into a 3-level ordinal variable using cut-points used previously [25]. Categories were as follows: (1) low anger expression (scores < 10); (2) moderate anger expression; and (3) high anger expression (scores ≥ 15). Dichotomous variables for each item of the anger scale were also created so that risk of violent behavior could be assessed for students who ever outwardly expressed anger (almost never versus sometimes, often and almost always combined). An overall negative life events score was defined as the sum of the number of life events that respondents experienced as negative [15]. Categories were determined *a priori *using same cut-points as previously described by Gudlaugsdottir et al [15]. Participants' age was treated as a continuous variable. Other variables were categorized as follows: college level (freshman, sophomore, junior, and senior); religious affiliation (Ethiopian Orthodox Christian, Protestant, Muslim and other); childhood residence (rural or urban); cigarette smoking (no, yes); alcohol consumption (no, yes); use of khat (no, yes); witnessing of parental violence as a child (no, yes).

Bivariate statistical analytical methods were initially used to explore frequency distributions of participants' socio-demographic and lifestyle characteristics information according to students' depression status. For continuous variables, group-specific means and standard error of the mean (SEM) were reported.

Logistic regression procedures were used to determine the risk of depression in relation to outward anger expression and history of violent behavior during the current academic year. Multivariable logistic regression models were constructed to calculate maximum likelihood estimates of odds ratios (OR) and 95% confidence intervals (95% CI) adjusted for confounders [32]. Confounders were defined as those covariates which altered unadjusted ORs by at least 10%. We considered the following covariates as possible confounders in these analyses: age, education level, religious affiliation, alcohol consumption, cigarette smoking, childhood residence, witnessing parental violence, and negative life events. All reported p-values are 2-tailed, and statistical significance was set at 0.05.

## Results

Some 23.6% (277 of 1,176) of students in this study population had symptoms consistent with some degree of depression during the current academic year. Only a small fraction of students had symptoms consistent with moderately severe (75, 6.4%) or severe (27, 2.3%) depression. These prevalence estimates of students with symptoms of depression are similar to those reported elsewhere [33]. Demographic and lifestyle characteristics of students with no symptoms of depression versus those with symptoms of depression are summarized in Table 1. Students with depressive symptoms were more likely to report khat use, alcohol consumption and cigarette smoking, though only the latter behavior reached statistical significance. Students with depressive symptoms were more likely to report witnessing parental violence as a child and to have 3+ negative life events during the academic year. Students with and without depressive symptoms were similar with respect to age, college level, religious affiliation, and childhood residence.

**Table 1:** Demographic and lifestyle characteristics according to symptoms of depression among study participants

**Characteristics**	Depression*		**P-value**
		
	**No (N = 899)** **n (%)**	**Yes(N = 277)** **n (%)**	
**Age (yr)**^α^	21.5 ± 0.1	21.6 ± 0.2	0.181
College Education Level**			
Freshman	345 (38.5)	111 (40.4)	0.824
Sophomore	179 (20.0)	48 (17.3)	
Junior	226 (25.2)	71 (25.6)	
Senior	146 (16.3)	45 (16.2)	
**Religion**			
Orthodox	489 (54.4)	144 (52.4)	0.194
Protestant	268 (29.8)	88 (31.8)	
Muslim	72 (8.0)	15 (5.4)	
Other	60 (6.7)	28 (10.1)	
**Childhood Residence**			
Rural	427 (47.9)	141 (51.5)	0.298
Urban	465 (52.1)	133 (48.5)	
**Khat chewing**			
No	613 (69.5)	176 (65.2)	0.104
Yes	269 (30.5)	94 (34.8)	
**Smoking**			
No	747 (88.0)	214 (81.4)	0.005
Yes	102 (12.0)	49 (18.6)	
**Alcohol Drinking**			
No	447 (52.5)	126 (48.1)	0.121
Yes	405 (47.5)	136 (51.9)	
**Witnessing Parental Violence**			
No	556 (75.0)	148 (65.5)	0.005
Yes	185 (25.0)	78 (34.5)	
**Negative Life Events**			
None	156 (17.4)	18 (6.5)	<0.001
1	98 (10.9)	20 (7.2)	
2	98 (10.9)	34 (12.3)	
3	112 (12.5)	47 (17.0)	
≥4	435 (48.4)	158 (57.0)	

^α^ Mean ± SEM

*No (Score < 9) and Yes (Score ≥ 10)

**Numbers/percentages may not add up to the total number due to missing data

The mean anger-out expression score was 12.9 (standard deviation 3.9) (table not shown). Overall, 67.8% of students were classified as having a moderate or high outward anger expression (797 of 1,176). Notably, 29.3% (344 of 1,176) of students were classified as having a high (anger-out score ≥ 15) level of outward anger expression. These values are consistent with reports from other populations [10,12,14].

Anger expression and reports of violent acts were both positively associated with the presence of depressive symptoms among students in our study populations (Table 2). Students with moderate outward anger expression (score: 11–14), were 2.03-times more likely to have depressive symptoms (OR = 2.03; 95% CI 1.40–2.95) as compared with those students classified as having low outward anger expression (score ≤ 10). Students with high (≥ 15) anger expression were 3.75-times more likely to have depressive symptoms as compared with their counterparts with low anger expression (OR = 3.75; 95% CI 2.58–5.44). Moderate (adjusted OR = 1.97; 95% CI 1.33–2.93) and high (adjusted OR = 3.23; 95% CI 2.14–4.88) outward anger remained statistically significantly associated with the presence of depressive symptoms after we controlled for confounders.

**Table 2:** Odds ratios (OR) and 95% confidence intervals (CI) of depression among study participants

**Characteristics**	**Depression**		**Unadjusted** **OR (95% CI)**	*Adjusted **OR (95% CI)**
			
	**No (N = 899)** **n (%)**	**Yes(N = 277)** **n (%)**		
**Anger Out Expression Score**				
8–10	330 (36.7)	49 (17.7)	1.00 (Reference)	1.00 (Reference)
11–14	348 (38.7)	105 (37.9)	2.03 (1.40–2.95)	1.97 (1.33–2.93)
≥15	221 (24.6)	123 (44.4)	3.75 (2.58–5.44)	3.23 (2.14–4.88)
**Violent Act During Current Year**				
No	441 (49.1)	96 (34.7)	1.00 (Reference)	1.00 (Reference)
Yes	458 (50.9)	181 (65.3)	1.82 (1.37–2.40)	1.12 (0.82–1.45)

*Separate models used for each variable

*Adjusted for College Level (Continuous), Witnessing Parental Violence (Yes/No), Negative Life Events (Continuous), and Smoking (No/Yes)

We also evaluated the extent to which, if at all, violent behavior was associated with symptoms of depression. As can be seen in table 2, in bivariate analyses, violent behavior was associated with depressive symptoms (unadjusted OR = 1.82; 95% CI 1.37–2.40); however, the association was greatly attenuated, and became statistically insignificant after adjustment for confounding variables (adjusted OR = 1.12; 95% CI 0.82–1.45).

Next we evaluated frequencies of different types of outward expression of anger (Table 3). Students in the present study were most likely to "verbally express their anger" to others (70.2% responded sometimes, often, or almost always); and were apt to "tell someone if annoyed" by them (66.2%) (Table 3). Students were less likely to report "striking out" when angered. The following specific outward expressions of anger were found to be statistically significantly associated with depressive symptoms: "slamming doors" (OR = 1.88; 95% CI 1.40–2.52), "arguing with others" (OR = 1.63; 95% CI 1.22–2.19), "striking out at whatever infuriates" (OR = 2.43; 95% CI 1.78–3.31), "saying nasty things" (OR = 2.06; 95% CI 1.52–2.78), and "losing temper" (OR = 1.78; 95% CI 1.34–2.41) (Table 4).

**Table 3:** Frequencies of anger-out behaviors according to depressive symptoms among study participants

	**Almost Never** **n (%)**	**Sometimes** **n (%)**	**Often** **n (%)**	**Almost Always** **n (%)**
**Type of Anger-Out Behaviors**				
***Express anger***	350 (29.8)	612 (52.0)	168 (14.3)	46 (3.9)
***Make sarcastic remarks***	770 (65.5)	307 (26.1)	71 (6.0)	28 (2.4)
***Slam doors***	675 (57.4)	385 (32.7)	87 (7.4)	29 (2.5)
***Argue with others***	649 (55.2)	421 (35.8)	68 (5.8)	38 (3.2)
***Strike out***	871 (74.1)	228 (19.4)	52 (4.4)	25 (2.1)
***Say nasty things***	801 (68.1)	316 (26.9)	39 (3.3)	20 (1.7)
***Lose temper***	666 (56.6)	377 (32.1)	92 (7.8)	41 (3.5)
***Tell if someone annoys***	398 (33.8)	446 (37.9)	196 (16.7)	136 (11.6)

**Table 4:** Adjusted odds ratios of anger-out behaviors according to depressive symptoms among study participants

	Adjusted* OR (95% CI)
**Anger-Out Behaviors**	
***Express anger***	1.15 (0.84–1.59)
***Make sarcastic remarks***	1.43 (1.06–1.92)
***Slam doors***	1.88 (1.40–2.52)
***Argue with others***	1.63 (1.22–2.19)
***Strike out***	2.43 (1.78–3.31)
***Say nasty things***	2.06 (1.52–2.78)
***Lose temper***	1.78 (1.34–2.41)
***Tell if someone annoys***	1.01 (0.79–1.39)

*Adjusted for College Level (Continuous), Witnessing Parental Violence (Yes/No), Violent Behavior (No/Yes), Negative Life Events (Continuous), and Smoking (No/Yes)

## Discussion and conclusion

The results of our study provide support for the hypothesis that high levels of outward anger expressions are significantly associated with symptoms of depression. In contrast, violent behavior was not significantly associated with symptoms of depression. These findings are in general concordance with results reported in other studies [10,34,35].

In this study, moderate and high anger-out expression scores were associated with a 2.18-fold and 3.38-fold increased risk of having depressive symptoms respectively. These results are generally consistent with findings reported by others. Goodwin, in a survey conducted among US youths[10], reported that outward anger expression was associated with an almost 3-fold increased risk (OR = 2.8; 95% CI: 2.5–3.1) of experiencing symptoms of depression among boys. Sperberg et al, in their study of 223 female college students noted that anger-out was positively associated with symptoms of depression (Pearson's Correlation Coefficient = 0.23; p < 0.01), and that the association remained significant even after adjusting for potential confounding variables [34]. Similarly, Lee and colleagues, in their study of Korean cancer patients, reported a positive association between outward anger expression and symptoms of depression (Pearson correlation coefficient = 0.240, p = 0.001) [11].

Our results and those of other investigators working in this area, however, are not consistent with findings from a recent study in Japan [14]. Kitamura and Hasui [14], in their study of 457 students reported that outward anger expression was not associated with depression (Pearson's correlation coefficient = 0.083). However, careful examination of results from their multivariate analysis suggested a statistically significant inverse association between outward anger expression and symptoms of depression (β = -0.163, p < 0.01).

The results of our study indicate that although violent behavior was associated (OR = 1.82; 95% CI 1.37, 2.40) with symptoms of depression in univariate analysis, the relationship was greatly attenuated (OR = 1.08; 95% CI 0.73, 1.60) when other covariates were included in multivariate models. Reports pertaining to association between violent behavior and symptoms of depression have been inconsistent. Salmon et al, in their study among 904 UK secondary school students reported that adolescents with violent behavior acts were 3.3-times more likely to report symptoms of depression (OR = 3.3; 95% CI 1.6–6.7) [7]. Similarly, in Finland, Kaltiala-Heino et al found that those who reported frequent violent acts were associated with a 2.8-fold increased risk (OR = 2.8; 95% CI 2.2–3.7) of experiencing symptoms of depression [6]. Inconsistencies across studies may be attributable to differences in population characteristics, type of study design, types of instruments used to assess violent behavior and symptoms of depression and variations in socio-cultural characteristics across study populations. Clearly more studies, particularly those that apply uniform sampling and assessment methods are warranted to move this area of literature forward.

Symptoms of depression, outward anger expression and violent behavior acts have been found to differ within cultural contexts and experiences [13,36,37]. More research is needed to identify the specific cultural characteristics of our study population and to deduce how certain cultural elements might influence students' outward expression of anger as a manifestation of depression.

Several limitations must be considered when interpreting the results of our study. First, the cross-sectional design of our study does not allow for conclusions to be formed about causality. For instance, although an association exists between negative life events and depressive symptomology, we cannot necessarily support the intuitive notion that the onset of depression spurs from negative experiences; perhaps depressed youth adopt a cognitive style in which they interpret all life events in a more negative light [38]. Second, our study population was exclusively comprised of young male participants who were currently attending college, thus conclusions cannot be generalized to all males (e.g. children, adults, college drop-outs), all college students (e.g. female), or other broader populations (e.g. all students in East Africa). Third, the instruments used to assess anger expression and symptoms of depression are not comprehensive. Our study exclusively dealt with Spielberger anger-out expression. Future studies should include other dimensions of anger expression such as anger-in and anger-control. Additionally, we used the PHQ-9, an instrument not previously validated in the present study population, to assess depressive symptoms. The PHQ-9 instrument, however, has been used widely to estimate prevalence of depressive symptoms in community-based settings around the world[39]. Investigators have reported that the measurement of depression in international contexts may be undertaken with similar instruments in different cultural settings, provided care is taken to ensure an adequate translation and validation of the cut-off score[40]. Along these lines, investigators have shown that the PHQ-9 can be used in the African context [41,42]. Adewuya and colleagues [41] in their study of Nigerian university students reported that the PHQ-9 is a valid and useful instrument in screening for depression in their settings. Additionally the PHQ-9 has been used successfully by Omoro and colleagues [42] in their study of Kenyan cancer patients. Fourth, the present analysis is limited in that we were not able to quantify the mediating and interactive effects of anger expression and depression on the occurrence of violent behavior. Finally, our study focused on violent behavior perpetrators and did not include victims of violence. Although the adverse health outcomes on victims of violent behavior has been well established, future studies that investigate the independent and joint associations of both perpetrators and victims of violent behavior and adverse social and mental health outcomes are needed.

In summary, outward anger expression was positively associated with symptoms of depression among male Ethiopia college students, a population that has been rarely studied, despite the high prevalence of violence in Ethiopia and other parts of Africa [8,43,44]. From a public health perspective, these data suggest that outward anger expression, perhaps more than violent behavior, may be useful as a means to identify students at increased risk of depression and depressive symptoms. Our findings expand the existing literature and underscore the global significance of links between outward expression, violence and depressive symptoms. The high prevalence of violence [8,44], depression [1] and outward anger expression [14] among male college students is particularly important given that they are likely to assume leadership positions important in influencing the future economic, political, and socio-cultural fabric of their respective communities. Further research should be conducted to better characterize community and individual level determinants of anger, violent behavior and depression among youths. Results from such research should then be used to develop school-based intervention programs and treatment programs designed to reduce violent behavior and promote appropriate anger expression skills among adolescents and young adults.

## Competing interests

The authors declare that they have no competing interests.

## Authors' contributions

Each author participated in the design, analysis, and interpretation of results. All authors contributed to the writing or editing of this manuscript.

## Pre-publication history

The pre-publication history for this paper can be accessed here:


